# MicroRNA expression profiles in human CD3^+^ T cells following stimulation with anti-human CD3 antibodies

**DOI:** 10.1186/s13104-017-2442-y

**Published:** 2017-03-14

**Authors:** Isabel Garcia Sousa, Manuela Maragno do Almo, Kelly Cristina Rodrigues Simi, Maryani Andressa Gomes Bezerra, Rosângela Vieira Andrade, Andréa Queiroz Maranhão, Marcelo Macedo Brigido

**Affiliations:** 1 Molecular Pathology Graduation Program, Medicine Faculty, University of Brasilia, Brasilia, Brazil; 2 Molecular Biology Graduation Program, Institute of Biological Sciences, University of Brasilia, Brasilia, Brazil; 3 Department of Cell Biology, Institute of Biological Sciences, University of Brasilia, Brasilia, Brazil; 4 Catholic University of Brasília, Brasilia, Brazil; 5 Institute for Immunology Investigation, A National Institute of Science and Technology, Brasilia, Brazil

**Keywords:** Recombinant antibody, Anti-CD3, miRNA, Immunoregulation, Immunosuppression

## Abstract

**Background:**

Anti-CD3 therapy can induce immunosuppression by several non mutually exclusive mechanisms that have been proposed to explain the therapeutic effect the administration anti-CD3 mAb, but its immunoregulatory mechanism is still not completely clear. In T cells, microRNAs (miRNAs) regulate several pathways, including those associated with immune tolerance. Here, we report changes in miRNA expression in T cells following treatment with anti-human CD3 antibodies. Peripheral blood mononuclear cells were cultured in the presence of the monoclonal antibody OKT3 or a recombinant fragment of humanized anti-CD3. Following these treatments, the expression profiles of 31 miRNA species were assessed in T cells using TaqMan arrays.

**Results:**

Eight of the tested miRNAs (miR-155, miR-21, miR-146a, miR-210, miR-17, miR-590-5p, miR-106b and miR-301a) were statistically significantly up- or down-regulated relative to untreated cells.

**Conclusions:**

Stimulation of T cells with anti-human CD3 antibodies alters miRNA expression patterns, including of miRNA species associated with immune regulatory pathways.

**Electronic supplementary material:**

The online version of this article (doi:10.1186/s13104-017-2442-y) contains supplementary material, which is available to authorized users.

## Background

CD3^+^ T cells play a major role in immune responses associated with autoimmune disease and organ transplantation. These cells form heterogeneous populations that can be distinguished based on molecular surface markers, and subsets of these markers can be used to denote various stages of T lymphocyte differentiation [1, 2]. Following activation by antigens and co-stimulatory signals, CD3^+^ CD4^+^ T cells orchestrate immune responses by differentiating into various effector T cell subsets, including Th1, Th2, Th17 and regulatory T cells [3, 4].

Clinical data have suggested that anti-CD3 therapy is a promising treatment option for autoimmune disease and organ transplantation. The mechanism of action underlying this therapy is not fully understood, and several non mutually exclusive mechanisms have been proposed to explain the therapeutic effect of the administration anti-CD3 mAb and the generation of Tregs (T regulatory cells) that seems to be associated to immunosuppression and immunological tolerance [5–8]. In vivo, T cells are stimulated by T cell receptors (TCRs), an integral component of which is CD3. In the presence of co-stimulatory signals, T cells differentiate into specific phenotypic subtypes. Several of these subtypes are involved in suppressing or terminating natural inflammatory signals. Hence, the clinical administration of anti-CD3 antibodies may interfere with or overcome natural TCR stimulation and therefore lead to the accumulation of suppressive T cell populations, including CD4^+^ CD25^+^ FOXP3^+^Tregs [9–11].

The anti-CD3 monoclonal antibody OKT3 (muromonab-CD3, Ortho Biotech) was the first monoclonal antibody (mAb) approved for clinical use in transplantation rejection therapy [12]. However, OKT3 is a murine monoclonal antibody that displays high toxicity in humans due to its heterologous nature and mitogenic activity. The effectiveness of long-term OKT3 therapy is hampered both by cytokine release syndrome and the presence of neutralizing antibodies [13]. Due to these clinical limitations, OKT3 was removed from the market in 2010 [14, 15]. Currently, a new generation of anti-CD3 therapy is being developed [16]. We have previously described a humanized anti-CD3 antibody fragment that displays less mitogenic activity than OKT3 [17] and may therefore be useful in modulating the immune system.

MicroRNAs (miRNAs) are small, non-coding RNA molecules that control many important cellular processes, including development, differentiation, survival, cell fate determination, and proliferation. Additionally, miRNAs have a pivotal role in immune cell functioning by controlling cytokine, chemokine, growth factor, cell adhesion molecule, and co-stimulatory molecule expression; antibody production; inflammatory mediator release and apoptosis induction [18–24].

Experimental ablation of miRNAs has demonstrated the importance of these molecules in T cell development, especially with regard to Tregs [25]. Therefore, tracking changes in miRNA profiles following stimulation with anti-CD3 antibodies may offer insights into stimulation of T cell transformation, reveal potential methods of programming differentiation, and produce valuable biomarker information. In this work, we measured changes in miRNA expression in anti-CD3 antibody-stimulated human T cells in vitro in the context of the peripheral blood mononuclear cell (PBMC) milieu. By comparing two different antibodies, a mAb and a recombinant antibody fragment, we unveiled changes in miRNA expression profiles that may be associated with T cell fate.

## Methods

### Donors

Peripheral blood was collected from five healthy individuals enrolled in this study (Additional file 1: Table S1). The study protocol was approved by the local ethics committee (CAAE: 32874614.4.0000.0030).

### Antibodies

The anti-CD3ɛ antibody Muromonab-CD3 (OKT3) was purchased from eBioscience (San Diego, CA, USA), and humanized antibody fragment (a fusion of a scFv and gamma 1 Fc) was produced in transfected CHO-K1 cells as previously described (FvFc version R) [17].

### Stimulation of PBMCs and T cell enrichment

Fresh PBMCs were isolated using Ficoll-Paque density gradient centrifugation (GE Healthcare, Sweden). Whole PBMCs were cultured in RPMI media (Invitrogen, Carlsbad, CA, USA) supplemented with 4 mM l-glutamine and 10% FBS in the presence or absence of soluble anti-CD3 antibodies. A total of 250 ng of antibody was applied to PBMCs at a concentration of 1 × 10^6^ cells/mL. After 72 h, CD3^+^ T cells were isolated by negative selection using magnetic beads according to the manufacturer’s instructions (Dynabeads^®^ Untouched™ Human T Cells Kit, Invitrogen).

### Flow cytometry

T cell subpopulations and the efficiency of T cell isolation from PBMCs were quantified by three-color staining using the following sets of mAbs: (1) anti-CD18 FITC (Becton–Dickinson, San Jose, CA, USA), anti-CD3 APC (eBioscience) and anti-CD4 PE (eBioscience) or (2) anti-CD18 FITC (BD), anti-CD3 APC (eBioscience) and anti-CD8 PE (eBioscience). Percentages of T cells (gated on CD18^+^ and CD3^+^) expressing CD4 or CD8 were measured by flow cytometry (Additional file 1: Table S2, Figure S1). All FACS data were acquired on a FACS Verse (BD) using BD FACSuite™ software (BD). Data were analyzed using FlowJo software version 10 (Tree Star, Ashland, OR, USA).

### RNA extraction

Total RNA, including small RNA species such as miRNAs, was extracted from T cells isolated after PBMC stimulation using a miRNeasy^®^ Mini Kit (Qiagen, Valencia, CA, USA). The extracted RNA was treated with TURBO™ DNase (Life Technologies, Grand Island, NY, USA) to eliminate genomic DNA. RNA integrity and purity were evaluated using a Bioanalyzer 2100 (Agilent Technologies Genomics, Santa Clara, CA, USA). All RNA samples used in this work had a RIN > 7.

### qPCR assays

qPCR assays were performed using an ABI Step One Plus Real-Time PCR System (Applied Biosystems, Austin, Texas, EUA). The 2^−ΔΔCt^ method was used to calculate mRNA or miRNA transcript levels (fold change). RT^2^ Profiler PCR Array Data Analysis software (SABiosciences, Frederick, MD, USA) was used for analysis. Three independent experiments were performed running in triplicates. For each sample, normalization was performed by subtraction of the median Cq values of treated and untreated samples for the normalization standards, that are specified in “miRNA profiling” section for miRNA and “Individual gene expression assays” section for genes. The p-values were calculated based on Student’s *t* test results of the replicate 2^−ΔΔCt^ values for each gene in the control samples and treatment samples. GraphPad Prism software version 6 and R package were used to make figures.

#### miRNA profiling

miRNA profiling was performed using TaqMan Arrays MicroRNA customized plates according to the manufacturer’s instructions (Applied Biosystems); 32 miRNAs were used without pre-amplification (Additional file 1: Table S3). Approximately 600 ng of total RNA extracted from T cells was utilized for cDNA synthesis, which was accomplished using a TaqMan^®^ MicroRNA Reverse Transcription Kit (Applied Biosystems). The miRNAs were then evaluated via qPCR using TaqMan^®^ Universal Master Mix II (Applied Biosystems) following the manufacturer’s instructions. RNU48 small non-coding RNA (snRNA) was used as an internal control for data normalization. miRNA data was deposited in GEO (Additional file 2).

#### Individual gene expression assays

Approximately 240 ng total RNA isolated from T cells following PBMC stimulation was utilized for cDNA synthesis using an RT^2^ First Strand Kit (Qiagen). Briefly, individual gene expression was measured using RT^2^ qPCR SYBRGreen/ROX MasterMix (Qiagen) following the manufacturer’s instructions. The following probes were used: *FOXP3*, *GITR*, *TBX21*, *STAT4*, *RORγt*, *STAT3*, and *GATA3* (Additional file 1: Table S4). The housekeeping gene *B2M* was chosen as an endogenous control.

## Results

### Specific miRNAs were differentially expressed in CD3^+^ T cells following stimulation with anti-human CD3 antibodies

**Fig. 1:**
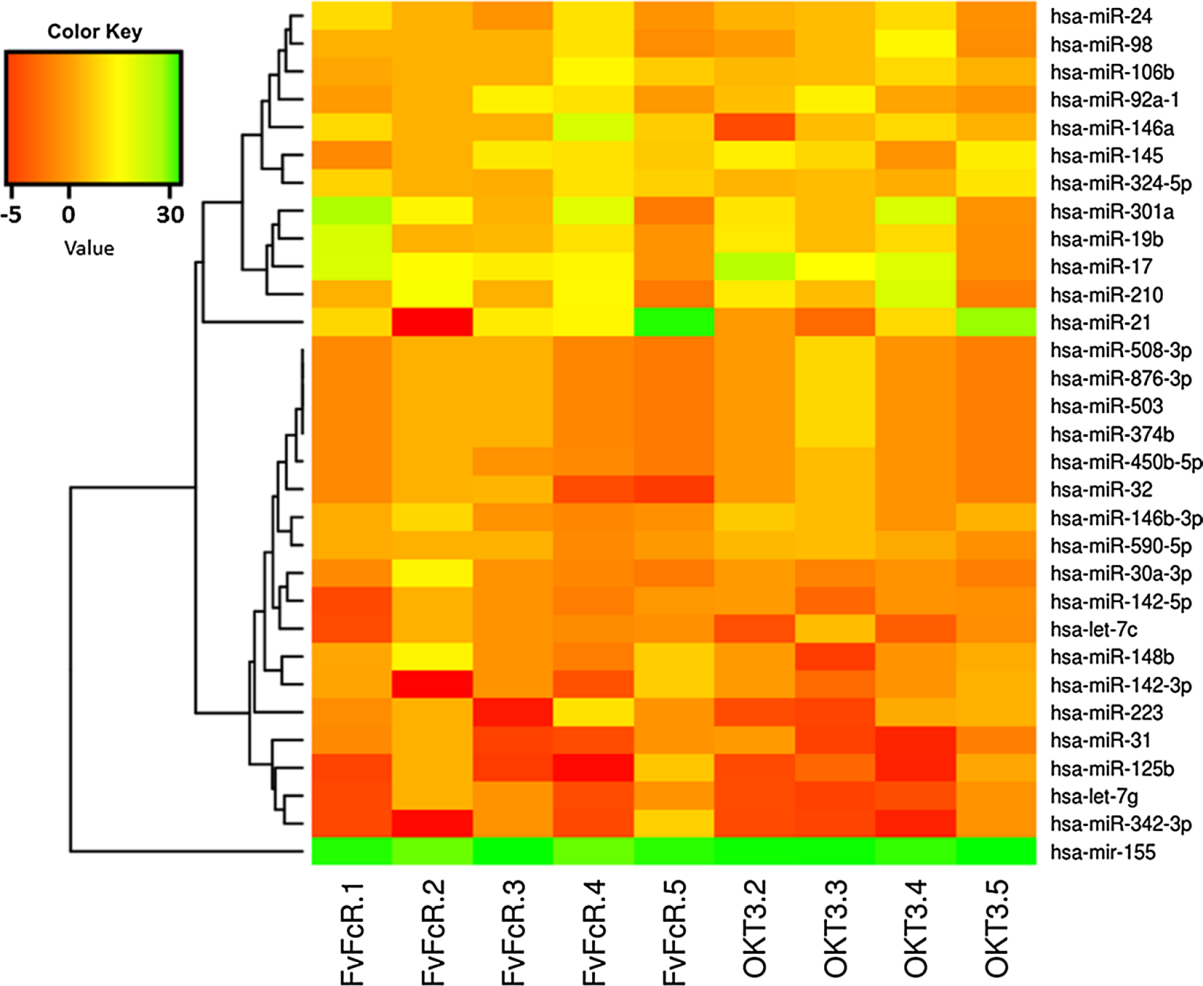
miRNA expression profile in T cells. Cluster analysis of 31 differentially expressed miRNAs in CD3^+^ T cells collected from healthy donors (n = 4–5). miRNAs that were up- or down-regulated in CD3^+^ T cells after CD3 stimulation. miRNA species are represented by* rows*, while samples are represented in* columns*. For each miRNA, *green* represents high expression, and *red* represents low expression relative to the average expression across all samples. This experiment was performed 72 h post stimulation, and the results are expressed as fold changes relative to levels in untreated T cells

To investigate how CD3 stimulation affected miRNA expression profiles, human PBMC were stimulated with anti-CD3 antibodies for 72 h. Then CD3^+^ cells were isolated and miRNA expression analyzed by quantitative PCR (qPCR). All 31 common miRNAs that were tested exhibited statistically significant changes in the samples from at least one donor when comparing cells stimulated with OKT3 or FvFcR to unstimulated cells (Fig. 1).

**Fig. 2:**
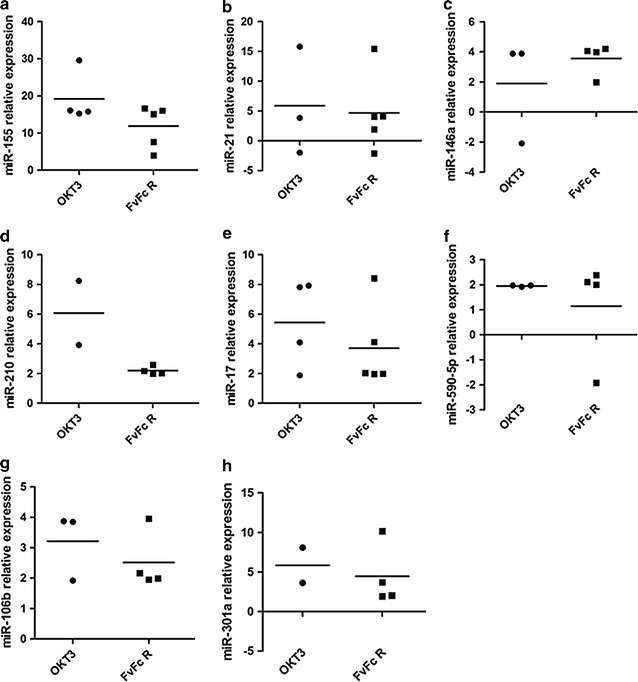
Quantitative analysis of changes in miRNA expression in CD3^+^ T cells following stimulation with anti-human CD3 antibody. qPCR was performed in triplicate 72 h post stimulation; the results are expressed as fold changes relative to levels in T cells (n = 5; p < 0.05). The presented miRNAs exhibited statistically significant changes in expression levels relative to untreated cells in 80% of the donors, for FvFcR treatment. RNU48 snRNA was used as an internal control for data normalization. **a** miR-155, **b** miR-21, **c** miR-146a, **d** miR-210, **e** miR-17, **f** miR-590-5p, **g** miR-106b, **h** miR-301a

The miRNA expression profiles displayed strong inter-donor variability. As they were the least variable, the CD3^+^ T cell expression profiles of eight distinct miRNAs, miR-155, miR-21, miR-146a, miR-210, miR-17, miR-590-5p, miR-106b and miR-301a, were further investigated (Fig. 2 and Additional file 1: Table S5).

miR-155 was consistently overexpressed following both antibody treatments: OKT3 seemed to induce stronger expression than FvFcR (Fig. 2a). miR-21 exhibited higher expression in T cells from most donors after stimulation with OKT3 and FvFcR antibodies compared to non-stimulated T cells (Fig. 2b). miR-31 was significantly down-regulated in a few donors (p < 0.05; Additional file 1: Figure S2). Anti-CD3 antibodies increased miR-146a expression in most PBMC donors, but FvFcR showed more consistent stimulation (Fig. 2c). The miR-210 expression profile was unique in exhibiting minimal variability between donors following FvFcR stimulation. FvFcR stimulated miR-210 less robustly than OKT3 (Fig. 2d). miR-17 was up-regulated in CD3^+^ T cells stimulated with either OKT3 or FvFcR (Fig. 2e). miR-590-5p expression increased in T cells following stimulation with OKT3 and FvFcR (Fig. 2f). All treatments induced up-regulation of miR-106b (Fig. 2g). OKT3 and FvFcR both up-regulated miR-301a expression in CD3^+^ T cells; OKT3 stimulation led to particularly strong expression (Fig. 2h). As indicated, there was a greater tendency toward up-regulated miRNA expression in stimulated versus unstimulated T cell subsets. Many of the evaluated miRNAs have been associated with the generation of Th17 and Treg populations as observed by other groups (Additional file 1: Table S3). Collectively, these data show that a limited number of miRNAs became differentially expressed in T cells following treatment with anti-human CD3 antibodies.

### Anti-human CD3 antibodies alter mRNA expression profiles in CD4^+^ T cells

**Fig. 3:**
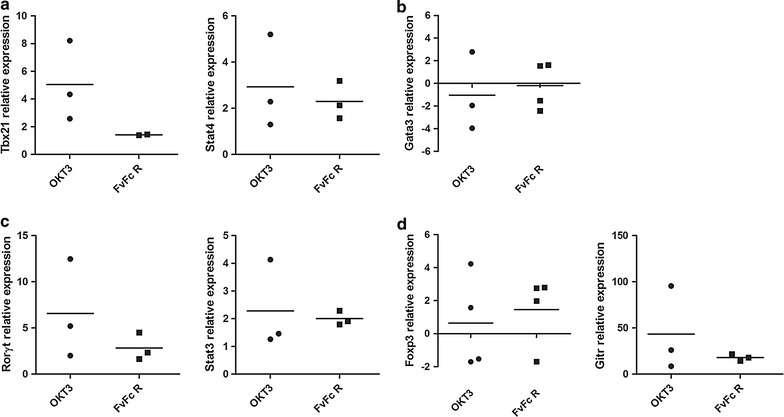
Effects of anti-CD3 stimulation on the expression of genes involved in CD4^+^ T cell differentiation. CD3^+^ T cells were isolated 72 h post stimulation, and mRNA was analyzed by qPCR to determine the expression levels of **a** *TBX21* and *STAT4*, **b** *GATA3*, **c** *RORγt* and *STAT3*, and **d** *FOXP3* and *GITR*. The data were normalized to the expression levels of the same genes in CD3^+^ T cells from untreated cultures. Representative results from three independent experiments are shown (n = 5; p < 0.05). B2M was used as an internal control for data normalization

Many of the miRNAs found to be differentially expressed have been associated with the generation of CD4^+^ T cells. To confirm the effects of anti-CD3 stimulation on T helper cell miRNA profiles, the expression patterns of genes considered typical markers of T helper cell differentiation were analyzed (Fig. 3; Additional file 1: Table S4). The expression levels of *TBX21*, *STAT4*, *RORγt* and *STAT3* were affected by stimulation with the anti-human CD3 antibodies FvFcR and OKT3 (Fig. 3a, c).

*TBX21* encodes T-bet, a transcription factor known to be involved in T helper cell commitment towards Th1 differentiation and TCD8 differentiation. *TBX21* mRNA expression was strongly induced after OKT3 stimulation (approximately an eightfold enhancement); recombinant FvFcR induced it to a lesser extent (Fig. 3a). In addition, the expression of *STAT4*, a gene that supports Th1 commitment, was induced; this induction suggests a pro-inflammatory response to anti-CD3 antibodies. Finally, antibody stimulation did not affect *GATA3* expression, which corroborates that T cells undergo Th1 polarization upon anti-CD3 treatment (Fig. 3b).

T helper cell differentiation may also induce Th17 and Treg phenotypes. Figure 2c shows the marked increase in *ROR*γt (RORC) and *STAT*3 expression that occurred following OKT3 stimulation. FvFcR also stimulated *ROR*γt expression but to a lesser extent. *STAT3*, a gene involved in Th17 cell signaling, was equivalently induced in all treatments (Fig. 3c). Additionally, the regulatory associated markers *FOXP3* and *GITR* were induced following all treatments (Fig. 3d); FvFcR was more effective at inducing *FOXP3*, while OKT3 more robustly induced *GITR*. These data suggest that stimulation with FvFcR influences genes that are associated Treg and Th17 cell phenotype.

## Discussion

In the present study, we characterized miRNA expression profiles in T cells following stimulation with anti-CD3 antibodies. Both a mouse monoclonal antibody and an Fc-bearing humanized antibody fragment were tested, and both could modulate miRNA expression in CD3^+^ T cells. Many of the miRNAs that changed in our study are enriched in conventional and regulatory CD4^+^ T cell populations, as well as in CD8^+^ T cells, and many are reported to play roles in regulating of immune response.

miR-155 is a well-studied miRNA that operates as a co-regulator of gene expression in multiple cell types to modulate the immune response [26]. miR-155 regulates cell growth and affects T cell polarization [27] by inducing a Th17/Treg bias in T helper cells [28]. Although activation by CD3/CD28 co-stimulation up-regulates miR-155 [29], it has also been shown that *FOXP3* controls miR-155 expression to maintain Treg proliferative activity [30]. Indeed, in vitro and in vivo experiments have shown that miR-155 deficiency reduces Treg populations in the thymus and periphery; however, Tregs from miR-155-deficient animals do not exhibit defects in suppressive function [28, 31]. Our data revealed a strong induction of miR-155 after antibody treatment, further supporting that this treatment could lead to the establishment of regulatory cells, a miR-155-sensitive population.

Despite the role of miR-155 in Treg survival, this molecule is still classified as proinflammatory, as it precisely regulates the levels of its targets to promote the immune response. Conversely, miR-146a and miR-21 are negative feedback regulators that mute the immune response [32]. It has been demonstrated that miR-21 acts as a positive indirect regulator of *FOXP3* expression; in contrast, miR-31 negatively regulates *FOXP3* expression by binding directly to its target site in the 3′UTR of *FOXP3* mRNA. Comparing miRNA expression profiles between human naïve CD4^+^ T cells with Tregs, miR-31 was found to be down-regulated in Treg cells, while miR-21 were found to be significantly up-regulated in this population [32]. miR-21 expression was induced after both antibody treatments, while miR-31 was consistently repressed by OKT3 treatment, and FvFcR treatment led to a variable response.

Naïve CD4^+^ T cells are reported to express low levels of miR-146a while these levels are increased in Tregs [33]. Rudensky and collaborators [34] reported that miR-146a is highly expressed in Treg cells and is critical for their function. The ablation of miR-146a impairs Treg function [28]. Moreover, miR-146a was shown to inhibit Th1 differentiation by interfering with *STAT4* signaling [34, 35]. Recently, the high level of miR-146a in naïve T cells was shown to enhance the suppressive effect triggered by Tregs [25]. In our data, both evaluated anti-CD3 antibodies induced miR-146a expression, but FvFcR stimulation seemed to produce a more homogenous response among donors. As such, FvFcR treatment may offer an important pathway for obtaining antibody-induced immune suppression.

miR-210 appears to bind to two targeting sites in the *FOXP3* mRNA 3′UTR to regulate human Treg differentiation [36]. In the present study, miR-210 expression was less stimulated by FvFcR than OKT3; as such, the recombinant antibody had less of an effect on this negative regulator of *FOXP3* expression relative to the mAb.

miR-301a inhibition in CD4^+^ T cells reduced IL-17 secretion and the expression of Th17 marker genes, such as *RORα*, *RORγt*, and *AhR*; however, this inhibition did not affect *TBX21* or *FOXP3* expression. miR-301a expression was particularly robust in Th17 cells both in vivo and in vitro. This strong expression suggests that miR-301a modulates Th17 development [37]. Furthermore, it has been reported that miR-301a inhibits *PIAS3*, a molecule known to interfere with the *STAT3* signaling pathway [28, 37]. Treating PBMCs with anti-CD3 antibodies led to a consistent increase in miR-301a levels among CD3^+^ T cells. This increase suggests a bias for Th17 polarization among naïve T helper cells.

Three of the up-regulated miRNA species (miR-106, miR-590-5p and miR-17) in the current study are also reported to be overexpressed in CD4^+^ T cells in multiple sclerosis (MS) patients [38–41]. Moreover, miR-106b and miR-590-5p exhibit higher expression in Tregs from MS patients compared to healthy controls [38]. miR-106b over-expression can silence two important effectors of the TGF-β signaling pathway: the cell cycle inhibitor CDKN1A and the pro-apoptotic gene *BCL2L11* [39], and miR-590-5p and miR-17 have been reported to target *TGFBRII*, which also affects TGF-β signaling [38, 40]. Moreover, miR-17 deficiency reduces *T*-*bet* and *IFN*-*γ* expression and promotes differentiation of Foxp3^+^ Tregs [40]. Therefore, this miRNA possesses a unique mechanism that reciprocally regulates Th1 and Treg generation [28, 38]. De Santis and collaborators suggested that disrupting the TGF-β signaling pathway, which is pivotal for Treg differentiation, would hamper immune suppressive activity, yielding the autoimmunity observed in MS. These data do not support the hypothesis that anti-CD3 stimulation would lead to Th17/Treg axis polarization, a process that relies on TGF-β signaling. However, we measured an entire CD3^+^ population, and it is possible that a fraction of this population expressed high levels of TGF-β-disrupting miRNAs and therefore differentiated into other types of effector cells (e.g., CD8^+^ or Th1 cells). Therefore, stimulation with anti-CD3 antibodies would affect a broad spectrum of differentiation pathways, including those responsible for producing effector and regulatory cells, and the balance between them would define the effective response. A more precise analysis of T cell subpopulations would offer a better definition of this balance.

In the current study, the miRNA expression changes produced following the stimulation of CD3^+^ T cells with anti-CD3 antibodies suggests that Th17/Treg polarization was associated with the induction of miR-146a, miR-21, and miR-155 expression; that together with miR-301 up-regulation, this process would result in a Treg bias. Conversely, the miR-17, miR-106, and miR-590-5p expression data suggest the disrupture of the TGF-β signaling. Therefore, it is possible that two different populations can be induced. To test this hypothesis, we measured the expression profiles of seven marker genes corresponding to the following CD3^+^ CD4^+^ subpopulations: Th1, Th2, Th17 and Treg. Th2 induction was not well represented because *GATA3* was not reproducibly modulated by either anti-CD3 antibody among donors. *TBX21* was primarily induced by OKT3 treatment, suggesting a Th1 response; however, OKT3 treatment has not been reported to result in the induction of Th1 cells in other studies [6]. However, the induction of *ROR*γt, *STAT3*, *FOXP3* and *GITR* suggest a bias toward the Th17/Treg axis. The subtle increase in *FOXP3* expression suggests the development of either CD4^+^ FOXP3^+^ or CD8^+^ FOXP3^+^ T cell populations, both of which are known suppressors of the immune response [42]. *GITR* expression is associated with the development of Tregs, even though *GITR* is also observed in CD8^+^ effector cells [43].

## Conclusions

Overall, in the present study, we have delineated the effects of two different anti-CD3 antibodies on human T cell miRNA expression. Both antibodies induced similar responses and exhibited individual fluctuations in induction and repression levels. As has been previously suggested for OKT3 [5], FvFcR could also induce Treg differentiation, as it was shown to increase the expression of miRNAs known to be positive regulators of *FOXP3* expression. Further investigation is warranted to dissect the precise roles of individual differentially expressed microRNAs in determining T cell fate. Overall, devoid of any co-stimulus, anti-CD3 stimulation in vitro promotes a clear change in the expression of a variety of miRNAs, including those operating in immune regulatory pathways. Moreover, the FvFcR molecule appears to be a promising immunomodulatory agent for the treatment of autoimmune diseases and organ transplantation.

## Additional files

**Additional file 1: Table S1.** Donors information. **Table S2.** Differentiation of CD3^+^ T cell subsets following stimulation with anti-human CD3 antibodies. **Table S3.** microRNAs chosen for array. **Table S4.** Genes chosen for cell phenotype marker. **Table S5.** microRNA ranking in CD3^+^ T cells according to p-value. **Figure S1.** Differentiation of CD3^+^ T cell subsets following stimulation with anti-human CD3 antibodies. **Figure S2.** Quantitative analysis of changes in miRNA expression in CD3^+^ T cells following stimulation with anti-human CD3 antibody.

**Additional file 2.** RAR compressed file containg miRNA and qPCR raw data in GEO format.

## Abbreviations

FvFc: single chain Fv fragment fused to a human IgG1 Fc
mAb: monoclonal antibody
miRNAs: microRNAs
PBMC: peripheral blood mononuclear cells
qPCR: quantitative PCR
TCR: T cell receptor
Tregs: T regulatory cells
