# Supplementary material for: MicroRNA expression profiles in human CD3+ T cells following stimulation with anti-human CD3 antibodies
Source: BMC Res Notes. 2017 Mar 14;10:124. doi: 10.1186/s13104-017-2442-y (PMC5351193; doi:10.1186/s13104-017-2442-y)
Supplement: Supplementary file 1 — Additional file 1: Table S1. Donors information. Table S2. Differentiation of CD3+ T cell subsets following stimulation with anti-human CD3 antibodies. Table S3. microRNAs chosen for array. Table S4. Genes chosen for cell phenotype marker. Table S5. microRNA ranking in CD3+ T cells according to p-value. Figure S1. Differentiation of CD3+ T cell subsets following stimulation with anti-human CD3 antibodies. Figure S2. Quantitative analysis of changes in miRNA expression in CD3+ T cells following stimulation with anti-human CD3 antibody. [file 13104_2017_2442_MOESM1_ESM.docx]

**Supplementary Information**

**Table 1.** Donors information.

| **Donors** | **Age** | **Gender** |
| --- | --- | --- |
| 1 | 30 | F |
| 2 | 28 | F |
| 3 | 30 | M |
| 4 | 28 | M |
| 5 | 22 | M |

Healthy PBMC donors; Age in years; F, female; M, male.

**Table 2.** Differentiation of CD3^+^ T cell subsets following stimulation with anti-human CD3 antibodies.

| **Anti-human CD3** | **% T CD4 cells** | **% T CD8 cells** |
| --- | --- | --- |
| **NT** | 56.8 | 39.6 |
| **OKT3** | 59.9 | 36.3 |
| **FvFcR** | 61.4 | 29.9 |

NT (non-treated). Human PBMCs were cultivated in the presence of recombinant antibodies, incubated with PE-conjugated anti-human CD4 or APC-conjugated anti-human CD8, and analyzed by flow cytometry. The percentages of the CD4^+^ and CD8^+^ subsets are shown.

**Table 3.** microRNAs chosen for array.

| **miRNAs** | **Cells involvement** | **References** |
| --- | --- | --- |
| hsa- miR-125b | - Naive CD4^+^ T cells  - Down regulated in CD4^+^ Treg cells | [3], [18], [44], [46] |
| hsa- miR-301a | Positively regulate CD4^+^ TH17 cells | [28], [37] |
| hsa- miR-374b | nTreg cells | [18] |
| hsa- miR-503 | Inhibit tumor progression and improve immune function | [49] |
| hsa- miR-590-5p | CD4^+^ Treg cells | [38] |
| hsa-let-7c | Down regulated in CD4^+^ Treg cells | [18] |
| hsa-let-7g | Down regulated in CD4^+^ Treg cells | [18] |
| hsa-miR-106b | Up regulated in CD4^+^ Treg cells | [18],[38], [39] |
| hsa-miR-142-3p | Negatively regulate CD4^+^ Treg cells | [18], [28] |
| hsa-miR-142-5p | Down regulated in CD4^+^ Treg cells | [18] |
| hsa-miR-145 | Negatively regulate CD4^+^ Treg cells | [28] |
| hsa-miR-146ª | - Positively regulate CD4^+^ Treg cells  - Higher expression In CD4^+^ Treg cells | [18], [25], [28], [32], [33], [34], [35], [44] |
| hsa-miR-146b-3p | Negative regulator of the innate immune response | [18] |
| hsa-miR-148b | Negative regulators of the innate response | [18] |
| hsa-mir-155 | CD4^+^ and CD8^+^ T cells | [18], [26], [27],[28],[29],[30],[31], [44] |
| hsa-miR-17 | - Negatively regulate CD4^+^ Treg cells  - Positively regulate CD4^+^ TH1 cells | [18], [28], [38], [40], [44] |
| hsa-miR-19b | - Positively regulate CD4^+^ TH1 cells  - CD8+ T cell subsets | [28], [45] |
| hsa-miR-21 | Positively regulate CD4^+^ Treg cells | [18], [32] |
| hsa-miR-210 | Negatively regulate CD4^+^ Treg cells | [28], [36] |
| hsa-miR-223 | Up regulated in CD4^+^ Treg cells | [18] |
| hsa-miR-24 | - Negatively regulate CD4^+^ Treg cells  - Expressed in Treg cells | [18], [28] |
| hsa-miR-30a-3p | CD8+ T cells | [45] |
| hsa-miR-31 | Negatively regulate CD4^+^ Treg cells | [18], [28] |
| hsa-miR-32 | Direct negative effect on the replication of retrovirus | [18] |
| hsa-miR-324 | Down-regulated in Tregs cells from peripheral blood samples of multiple sclerosis (MS) patients | [48] |
| hsa-miR-342-3p | Down regulated in CD4^+^ Treg cells | [18] |
| hsa-miR-450b-5p | Suppressed by TGF-β1 | [50] |
| hsa-miR-508-3p | Renal cell carcinoma (RCC) | [51] |
| hsa-miR-876-3p | Decreased in PBMCs from MS patients | [48] |
| hsa-miR-92 | CD4^+^ and CD8^+^ T cells | [18], [45], [47] |
| hsa-miR-98 | Down-regulated in peripheral blood of patients with all MS subtypes | [48] |

**Table 4.** Genes chosen for cell phenotype marker.

| **Genes** | **Cell involvement** | **References** |
| --- | --- | --- |
| TBX21 | Transcription factor involved in the regulation of developmental processes in Th1 cells | [37] |
| STAT4 | Transcription factor involved in development of Th1 cells from naive CD4+ T cells | [35] |
| GATA3 | Transcription factor involved in induces the differentiation of Th0 cells towards this Th2 cell subtype while suppressing their differentiation towards Th1 cells | [42] |
| RORC | Transcription factor involved in differentiation into proinflammatory Th17 cells | [37] |
| STAT3 | Transcription factor essential for the differentiation of the Th17 cells | [28] |
| FOXP3 | It is a master regulator of the regulatory pathway in the development and function of regulatory T cells | [42] |
| GITR | This receptor has been shown to have increased expression upon T cell activation, and it is thought to play a key role in dominant immunological self-tolerance maintained by CD25^+^CD4^+^ regulatory T cells | [43] |

**Table 5.** microRNA ranking in CD3^+^ T cells according to p-value.

| **OKT3** | | | **FvFcR** | | |
| --- | --- | --- | --- | --- | --- |
| **miRNA** | **P-value** | **Fold change** | **miRNA** | **P-value** | **Fold change** |
| **Donor 1** | | | | | |
|  |  |  | mir-155 | **0.000000** | 16.66 |
|  |  |  | miR-21 | **0.000000** | 4.12 |
|  |  |  | miR-17 | **0.000000** | 8.42 |
|  |  |  | miR-146a | **0.000001** | 4.20 |
|  |  |  | miR-106b | **0.000002** | 2.17 |
|  |  |  | miR-301a | **0.000013** | 10.16 |
|  |  |  | miR-210 | **0.000363** | 2.60 |
| **Donor 2** | | | | | |
| miR-17 | **0.000021** | 7.92 | miR-155 | **0.000023** | 4.03 |
| miR-155 | **0.000024** | 15.78 | miR-301a | **0.000230** | 1.92 |
| miR-210 | **0.000033** | 3.92 | miR-17 | **0.000265** | 2.04 |
| miR-301a | **0.000036** | 3.63 | miR-21 | **0.001588** | -2.12 |
| miR-106b | **0.000191** | 1.92 | miR-210 | **0.002740** | 2.18 |
| miR-146a | **0.000226** | -2.09 |  |  |  |
| **Donor 3** | | | | | |
| miR-155 | **0.000001** | 16.07 | miR-155 | **0.000000** | 16.02 |
| miR-17 | **0.000008** | 4.10 | miR-106b | **0.000000** | 1.99 |
| miR-21 | **0.000019** | -1.99 | miR-17 | **0.000001** | 4.13 |
|  |  |  | miR-21 | **0.000001** | 4.05 |
|  |  |  | miR-146a | **0.000011** | 1.99 |
|  |  |  | miR-301a | **0.000019** | 2.04 |
|  |  |  | miR-210 | **0.000110** | 2.01 |
| **Donor 4** | | | | | |
| miR-155 | **0.000000** | 15.24 | miR-155 | **0.000000** | 7.58 |
| miR-146a | **0.000001** | 3.89 | miR-301a | **0.000003** | 3.71 |
| miR-17 | **0.000002** | 7.81 | miR-146a | **0.000063** | 4.07 |
| miR-301a | **0.000003** | 8.09 | miR-106b | **0.000072** | 1.95 |
| miR-21 | **0.000004** | 3.85 | miR-21 | **0.000150** | 1.91 |
| miR-106b | **0.000009** | 3.87 | miR-17 | **0.000307** | 1.96 |
| miR-210 | **0.000083** | 8.24 | miR-210 | **0.000329** | 1.98 |
| **Donor 5** | | | | | |
| miR-21 | **0.000018** | 15.81 | miR-21 | **0.000003** | 15.45 |
| miR-155 | **0.000023** | 29.58 | miR-155 | **0.000006** | 15.13 |
| miR-106b | **0.000033** | 3.85 | miR-17 | **0.000010** | 1.98 |
| miR-146a | **0.000049** | 3.89 | miR-106b | **0.000014** | 3.95 |
| miR-17 | **0.000248** | 1.88 | miR-146a | **0.000052** | 3.99 |
| miR-301a | **0.000841** | 2.04 |  |  |  |

P-values are shown for the miRNAs that were statistically significantly up- or down-regulated between treated and untreated cells. (n = 4-5; p < 0,05).

**
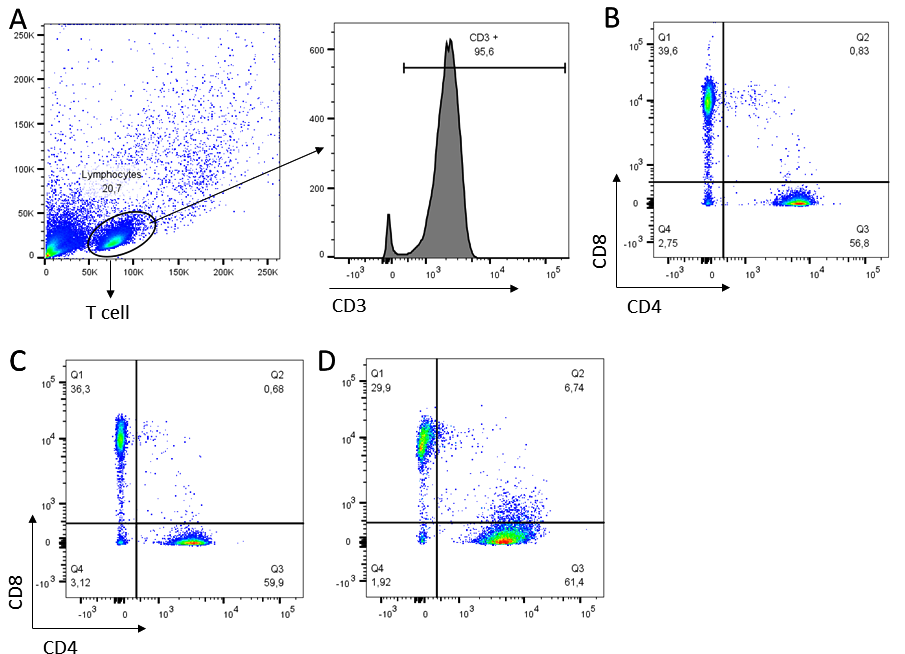
**

**Fig. 1.** Differentiation of CD3^+^ T cell subsets following stimulation with anti-human CD3 antibodies. The percentages of peripheral T cells, CD4^+^ T cells and CD8^+^ T cells in PBMCs from healthy volunteers were analyzed using flow cytometry after different stimulation conditions. (A) Unstimulated cells. The gated population of unstimulated lymphocytes is marked in the scattering plot. (B) Percentage of CD3^+^ cells gated in A. CD4 and CD8 labeling of cells stimulated with (C) OKT3 or (D) FvFcR.

**
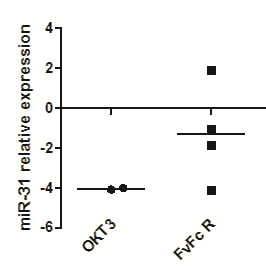
**

**Fig. 2.** Quantitative analysis of changes in miRNA expression in CD3^+^ T cells following stimulation with anti-human CD3 antibody. qPCR was performed in triplicate 72 h post stimulation; the results are expressed as fold changes relative to levels in T cells (n = 4-5; p < 0,05). RNU48 snRNA was used as an internal control for data normalization.
